# Feasibility and safety of integrating mass drug administration for helminth control with seasonal malaria chemoprevention among Senegalese children: a randomized controlled, observer-blind trial

**DOI:** 10.1186/s12936-023-04784-z

**Published:** 2023-11-13

**Authors:** Muhammed O. Afolabi, Doudou Sow, Schadrac C. Agbla, El Hadji Babacar Fall, Fatimata Bintou Sall, Amadou Seck, Isaac Akhénaton Manga, Ibrahima Marietou Mbaye, Mor Absa Loum, Baba Camara, Diatou Niang, Babacar Gueye, Doudou Sene, Ndéye M’backé Kane, Boubacar Diop, Awa Diouf, Ndéye Aida Gaye, Marie Pierre Diouf, Aminata Colle Lo, Brian Greenwood, Jean Louis A. Ndiaye

**Affiliations:** 1 London School of Hygiene & Tropical Medicine, London, UK; 2 Université Gaston Berger de Saint-Louis, Saint-Louis, Senegal; 3 University of Liverpool, Liverpool, UK; 4 Université de Thies, Thies, Senegal; 5 Université Cheikh Anta Diop, Dakar, Senegal; 6 Saraya Health Centre, Saraya, Senegal; 7 Ministry of Health and Social Action, Dakar, Senegal

**Keywords:** Falciparum malaria, Soil-transmitted helminthiasis, Schistosomiasis, Co-infection, Integrated control strategy

## Abstract

**Background:**

The overlap in the epidemiology of malaria and helminths has been identified as a potential area to exploit for the development of an integrated control strategy that may help to achieve elimination of malaria and helminths. A randomized, controlled, observer-blind trial was conducted to assess the feasibility and safety of combining mass drug administration (MDA) for schistosomiasis and soil transmitted helminths (STH) with seasonal malaria chemoprevention (SMC) among children living in Senegal.

**Methods:**

Female and male children aged 1–14 years were randomized 1:1:1, to receive Vitamin A and Zinc on Day 0, followed by SMC drugs (sulfadoxine-pyrimethamine and amodiaquine) on Days 1–3 (control group); or praziquantel and Vitamin A on Day 0, followed by SMC drugs on Days 1–3 (treatment group 1); or albendazole and praziquantel on Day 0, followed by SMC drugs on Days 1–3 (treatment group 2). Safety assessment was performed by collecting adverse events from all children for six subsequent days following administration of the study drugs. Pre- and post-intervention, blood samples were collected for determination of haemoglobin concentration, malaria microscopy, and PCR assays. Stool samples were analyzed using Kato-Katz, Merthiolate-iodine-formalin and PCR methods. Urine filtration, PCR and circulating cathodic antigen tests were also performed.

**Results:**

From 9 to 22 June 2022, 627 children aged 1–14 years were randomized into the three groups described above. Mild, transient vomiting was observed in 12.6% (26/206) of children in treatment group 2, in 10.6% (22/207) in group 1, and in 4.2% (9/214) in the control group (p = 0.005). Pre-intervention, the geometric mean value of *Plasmodium falciparum* parasite density was highest among children who received albendazole, praziquantel with SMC drugs. Post-intervention, the parasite density was highest among children who received SMC drugs only. Children who received praziquantel and SMC drugs had a lower risk of developing severe anaemia than their counterparts who received SMC drugs alone (OR = 0.81, 95% CI 0.13–5.00, p = 0.63).

**Conclusions:**

Integration of MDA for helminths with SMC drugs was safe and feasible among Senegalese children. These findings support further evaluation of the integrated control model.

*Trial registration*: The study is registered at Clinical Trial.gov NCT05354258.

## Background

Malaria is a leading cause of the high rate of child mortality that persists in sub-Saharan Africa (SSA) [1]. The World Health Organization (WHO) reported an estimated 234 million cases of malaria across SSA in 2021, representing about 95% of the global cases, with more than a half of the global malaria deaths occurring in four SSA countries [2]. The 2023 WHO global report on neglected tropical diseases (NTD) indicated that 584 million people living in SSA, accounting for over 35% of the global burden, require protection against NTDs [3]. In these African countries, co-infection of malaria with NTDs, such as schistosomiasis and soil-transmitted helminths (STH), is prevalent in vulnerable populations, especially among pre-school and school-age children. Geographical and host factors contribute to the co-existence of these major infectious diseases of global public health importance. The geographical overlap of *Plasmodium falciparum,* schistosomes and STH in SSA creates an opportunity for the development and implementation of an integrated control approach that could help to achieve the WHO targets of eliminating malaria in at least 13 countries between 2016 and 2030, and NTDs by 2030. This approach received a boost from the 2022 Kigali Declaration, in which global stakeholders committed USD 4.25 billion to exploit the epidemiological features shared by malaria and NTDs in order to end the two interconnected diseases by 2030 [4]. However, limited empirical evidence exists on the feasibility and safety of integrating or optimizing currently available control programmes for malaria and NTDs.

As a prelude to developing an integrated model for the control and elimination of co-infection with *P. falciparum, Schistosome* spp and STH, the authors conducted an evidence synthesis of 55 published articles on malaria-helminth co-infections to establish the burden of these mixed infections among children living in endemic countries [5]. To complement these findings, a geospatial analysis of malaria and helminth datasets was undertaken to identify transmission hotspots, that is areas where there is currently a high frequency of co-infections or where re-emergence of co-infections is likely to occur [6]. Using improved diagnostic methods, the authors also carried out two population-based surveys to establish the true estimates of the burden and the risk perception of malaria-helminth co-infections among children in an area of Senegal where both malaria and helminth infections are endemic [7, 8].

The establishment of an effective programme for delivering Seasonal Malaria Chemoprevention (SMC) with sulfadoxine-pyrimethamine and amodiaquine (SPAQ) to more than 40 million children resident in the Sahel and sub-Sahel areas, where malaria transmission is highly seasonal, and where helminth infections are prevalent, provides an opportunity for combining SMC with mass drug administration (MDA) programmes for prevention of schistosomiasis and STH. However, before a recommendation for combining MDA with anthelminthic drugs (praziquantel and albendazole) for schistosomiasis and STH with SMC drugs can be made, it is important to show that this would be safe and well tolerated by pre-school and school-age children and that the incidence of side effects would not be significantly higher in children who received the combined SMC and anthelminthic drugs than in those who received SMC drugs alone.

The findings of an intervention study (ClinicalTrials.gov NCT05354258) [9] which assessed the feasibility and safety of integrating MDA for schistosomiasis and STH with SMC among pre-school and school-age children living in a malaria-helminth co-endemic community in Senegal, are reported in this paper. The impact of combining antihelminth drugs administration with SMC on anaemia, malaria and schistosomiasis was also assessed.

## Methods

### Overall study design and study area

**Fig. 1:**
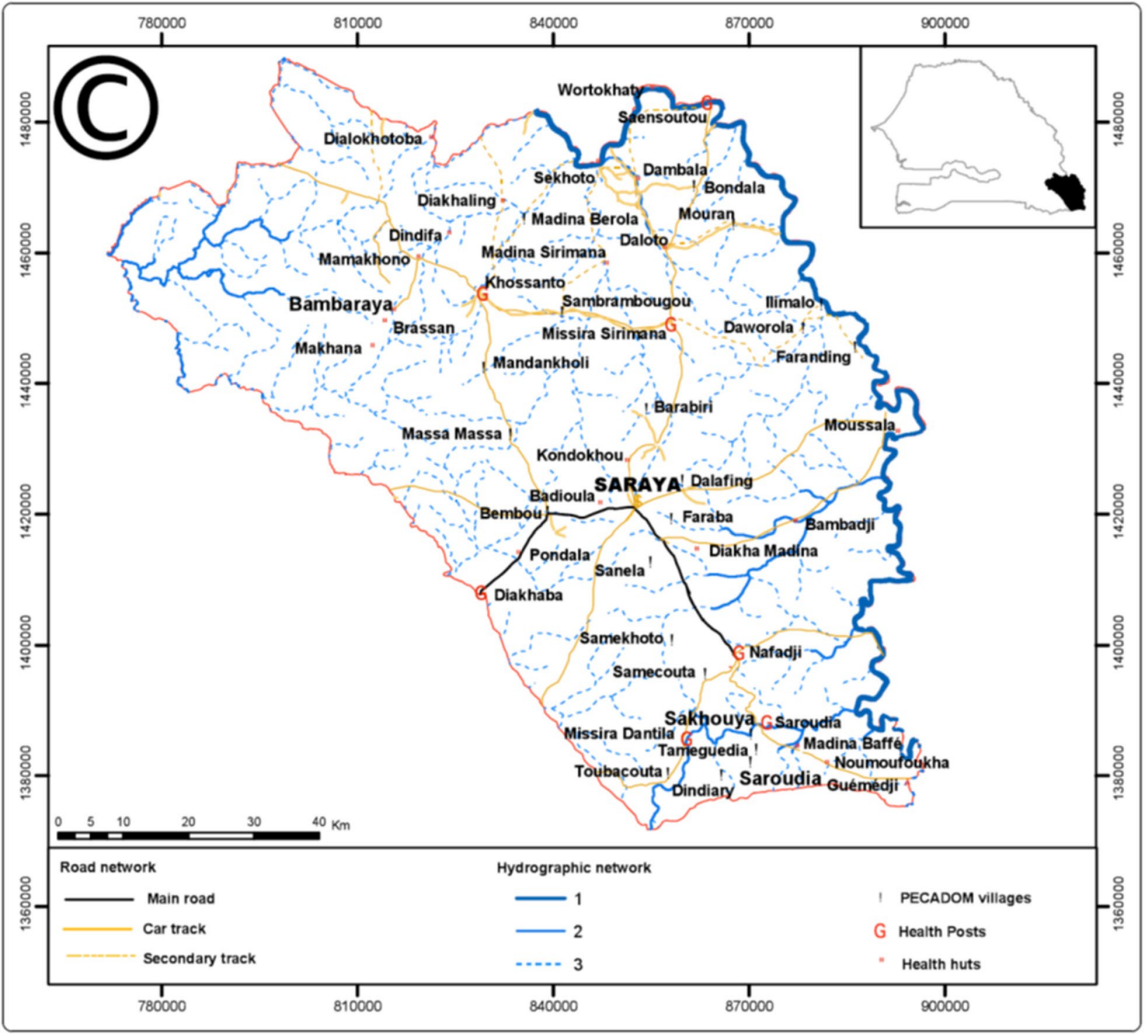
Map of Senegal showing the study site. Source: Ndiaye et al. Malaria Journal 2013, 12:240 http://www.malariajournal.com/content/12/1/240

This was a randomized, controlled, observer-blind trial conducted among pre-school and school-age children in the Saraya district of Kedougou region, southeast Senegal (Fig. 1). The characteristics of the study population and setting have been reported previously [9]. Briefly, Saraya is about 800 km from Dakar, the capital city of Senegal, and has an estimated population of 61,756 (2019 population census). Over 70% of the population live more than 5 km from a health facility. Subsistence farming is the main occupation of Saraya residents. Malaria and helminths are endemic in the district [10–13]. This trial was conducted in collaboration with the national malaria control programme and NTD programme of the Senegal Ministry of Health and Social Action.

Community engagement meetings were organized prior to the commencement of the study to explain the nature of the study to the parents/caregivers of potentially eligible children. During these meetings, the study team explained the need for this study, using a simple picture to illustrate the co-administration of SMC and anthelminthic drugs, the study rationale and informed consent procedure, and the risks and benefits of allowing their children to participate in the study. After these meetings, research staff identified parents/caregivers of potential study participants to explain the study to them in a more individual basis.

### Recruitment and randomization

Based on the findings of a population-based survey conducted across 18 villages in Saraya district in the preceding year [7], six villages with highest prevalence of malaria, schistosomiasis and STH were identified. Next, a census of potentially eligible children in the identified villages was conducted. In these villages, male and female children aged 1–14 years were recruited into this study. The parents/caregivers provided written informed consent and children aged ≥ 12 years who gave positive assent to join the trial (in line with legal regulations in Senegal), and who had been resident in the study area for at least 6 months, were enrolled into the study. Exclusion criteria included children with an acute illness at the time of drug administration; those known to be HIV positive and receiving cotrimoxazole prophylaxis; those who had received a dose of either sulfadoxine-pyrimethamine (SP), amodiaquine (AQ), albendazole (ALB) or praziquantel (PZQ) during the previous 6 months; or had a known allergy to any of SP, AQ, ALB or PZQ. Eligible children were randomized in blocks of 3, using a table of random numbers and stratified by age groups, into one of three groups in a ratio of 1:1:1 as follows: the first group, which served as a control arm, received Vitamin A and Zinc on Day 0, followed by SMC drugs (SP + AQ, known as SPAQ) on Days 1–3; the second group received praziquantel and Vitamin A on Day 0, followed by SMC drugs on Days 1–3; and the third group received albendazole (ALB) and praziquantel (PZQ) on Day 0, followed by SMC drugs on Days 1–3.

### Study procedures

One day before the planned start date of the study, written informed consent was obtained, and a pre-labelled stool collection cup was given to the parents/caregivers of eligible children. The parents/caregivers were encouraged to collect a stool sample from their children and to keep this safely for the research team to collect the following day. On the start day of the study (Day 0), after a research staff had confirmed that the parents had provided informed consent and the older children assent, a purpose-designed electronic questionnaire was administered to the parents/caregivers. The questionnaire collected information on items such as socio-demographic status, health and residence characteristics, history of de-worming and malaria treatment. The height/length (cm) and weight (kg) of each child participant were measured and the anthropometric indices height-for-age, weight-for-age, weight-for-height, and body mass index, were calculated using the WHO AnthroPlus software (www.who.int/growthref/tools/en/). The global-positioning system (GPS) coordinates of each study participant’s household was measured with a hand-held GPS device.

Prior to the administration of the study drugs, a finger-prick blood sample was collected from each child for malaria microscopy, and a blood spot filter paper sample was collected for DNA extraction and PCR amplification for species determination. Freshly voided urine and stool samples were also collected from all study children. The administration of the study drugs to the children and their subsequent safety assessments are described below. A post-intervention survey was conducted 5 months after co-administration of the study medications to assess the effectiveness of the combined treatment approach. Blood, urine and stool samples were collected again from all study participants during the post-intervention survey.

### Study drugs

The anthelminthic drugs used in this trial were the WHO-approved medications for MDA of schistosomiasis and STH control, namely PZQ and ALB, respectively. The SMC drugs given were SP and AQ. The SMC drugs were obtained from the SMC implementation unit of the Senegal Ministry of Health, and albendazole and praziquantel were obtained from the NTD control programme of the Senegal Ministry of Health. Vitamin A and Zinc supplements were used as control drugs, so that the children randomized to all study arms received three drugs. Vitamin A and Zinc supplements have not been shown to affect the primary and secondary outcomes of this study [14, 15]. Vitamin A was given as a liquid, oil-based preparation of retinyl palmitate acetate, obtained from UNICEF, Senegal office. The zinc preparation was a citrus-flavoured tablet containing 25 mg Zinc in the form of zinc sulfate and was procured from Biolectra Zink; Hermes Arzneimittel GmbH, Munich, Germany. The doses of SP and AQ, ALB, PZQ, Vitamin A and zinc supplement were based on the child’s age and weight (Table 1).

**Table 1:** Doses of study medications

	Children aged 12–59 months	Children aged 5–14 years
SP tablet (500 mg + 25 mg)	A full tablet as a single dose on the 1st day of the SMC cycle	Two tablets as a single dose on the 1st day of the SMC cycle
AQ tablet (153 mg base)	A full tablet as a single daily dose for 3 consecutive days of the SMC cycle	Two tablets as a single daily dose for 3 consecutive days of the SMC cycle
	Children aged 12–24 months	Children aged greater than 2 years and up to 14 years
ALB tablet (200 or 400 mg depending on age)	A half dose of albendazole 200 mg	A single dose of albendazole 400 mg
	Children aged 1–14 years	
PZQ tablet (600 mg)	Praziquantel based on their body weight 40 mg/kg	
Vitamin A (200,000 IU)	Children aged 1–14 years	
	200,000 IU Vitamin A	
Zinc (40 mg/kg)	Zinc supplement based on their body weight 40 mg/kg	

### Study drug administration

The anthelminthic and SMC drugs were only available in tablet form. Hence, to facilitate administration, the tablets were crushed into granules, and a teaspoonful of granulated table sugar was added to the crushed drugs. The study drugs were administered to the children by a trained pharmacist in the presence of their parents/caregivers. The anthelminthics (PZQ and ALB) were administered to the children randomized to receive them 24 h before SMC drugs (SPAQ) were administered as part of the annual SMC campaign. A 10-min interval separated the administration of albendazole and praziquantel in the study children randomized to receive these drugs. Parents were encouraged to provide their children with a carbohydrate-rich meal before the administration of the study drugs. If a child vomited any of the study drugs, a 30-min period was allowed before re-administration, but if vomiting occurred a second time, the treatment on that occasion was not considered part of the study. A simple, user-friendly recording tool was used to capture the drug administration for each child participant. To ensure objectivity and minimize bias in safety assessment and reporting, the staff who conducted safety assessment for the study participants were separate from those who gave the study drugs. Children who started the treatment but could not be found at home after reasonable efforts were excluded from the study.

### Safety assessment

Trained field workers visited all enrolled children daily at home starting from the evening of day 0 until 3 days after completion of the first cycle of SMC, using a purpose designed electronic diary card which recorded the presence of any side effects, including fever, vomiting, diarrhoea. In addition, all study participants who presented to the outpatient or emergency departments of Saraya health centre or health posts in the study villages were evaluated for malaria and any serious adverse events (SAE) that might have been related to administration of the study drugs. Trained research assistants visited each child 1 month after the first round of SMC cycle to check that there had been no severe reactions to the previous treatment, and if this was not the case, gave the next round of treatment. When a possible drug related SAE was reported to the study staff, the case was investigated by a Local Safety Monitor who was an experienced physician based in Senegal. The Local Safety Monitor reported the findings to the Sponsor of the study. Data obtained from all inpatient records for study children admitted to the hospital for at least 24 h within 1 month of SMC administration were evaluated for severity and a possible relationship to administration of the study drugs using standard criteria [16].

### Laboratory methods

Haemoglobin concentrations were measured using the HemoCue^®^ Hb 801 photometer, and anaemia was defined as an haemoglobin (Hb) concentration < 11.0 *g*/dl 10–10.9 *g*/dl as mild anaemia, 7–9.9 *g*/dl as moderate anaemia, and < 7 *g*/dl as severe anaemia [17]. Thick and thin blood films were prepared following standard operational procedures. Blood films were examined microscopically following standard procedures [18]. Slides were considered positive when asexual forms and/or gametocytes of any *Plasmodium* species were observed. Two experienced microscopists read all slides independently. Malaria parasite density per μl of blood was determined by counting the number of parasites per 200 leukocytes and multiplying by an average value of white blood cell count, considered to be 8000/μl. Parasitaemia was classified as low (≤ 500 parasite/μl of blood), moderate (501–5000 parasites/μl of blood) or high (> 5000 parasites/μl of blood) [19].

A freshly voided urine sample was collected from each study participant into a pre-labelled plastic container with a screw cap. The urine filtration test was used to quantify *Schistosoma haemotobium*, as described by Cheesbrough [18]. In addition, parallel testing for schistosome circulating cathodic antigens (CCA) in urine was performed [20]. Duplicate smears were prepared for each stool sample using a 41.7 mg Kato-Katz template. Each slide was allowed to clear for 30 min, and then examined by an experienced technician at 100 × total magnification within one hour of preparation to avoid missing hookworm eggs, and to determine the egg counts for *Schistosoma mansoni* and STH. The intensity of the helminth infection was categorized, as described by Cheesbrough [18]. Quality control was performed by re-examining at least 10% of randomly selected blood slides, urine filters and Kato-Katz smears by an experienced independent laboratory scientist. A multiplex PCR assay was used for simultaneous detection of mixed infections of helminths [21, 22]. Dried blood spots were analyzed for malaria using PCR methods, as described by Rougemont et al.[23].

### Merthiolate-iodine-formalin (MIF) technique for detection of intestinal protozoa

The traditional fixation method, MIF technique [24], was used to further examine the stool samples. The 2 × 24 Copro-Duo Kit (RAL Diagnostics, France) was used to highlight protozoan cysts, schistosome eggs and unfertilized *Ascaris* eggs. Stool samples were added to cryotubes to which mercurothiolate, iodine and formalin (MIF) solution (4 drops of Lugol in 15 µl of MIF) was added. The samples were stored at + 4 ℃, smears prepared and examined for parasite eggs using 10 × and 40 × microscope objectives.

### Study endpoints

The primary trial endpoint was safety, measured as the incidence of adverse events related to study medications during a 6-day follow-up period after randomization to each treatment arm (starting from day 0 of administration of anthelminthic drugs to 3 days after completion of the first cycle of SMC drugs. Unsolicited adverse events were recorded during a 30-day passive surveillance period after each SMC cycle, and SAEs throughout the study period. The following secondary endpoints were assessed before and after co-administration of SMC with anthelminthic drugs (i.e. 1 month after completion of the last SMC cycle which corresponded to the end of malaria transmission season: (i) the prevalence and intensity of *Plasmodium* spp. infection (ii) the prevalence and intensity of *Plasmodium*-helminth co-infection, (iii) the prevalence and intensity of each of the STHs (hookworm, *Ascaris lumbricoides, Trichuris trichiura,* and *S. mansoni*) infections; (iv) the prevalence and intensity of *S. haematobium* infection, and (v) the prevalence of anaemia and mean haemoglobin concentration.

### Sample size determination

A minimum sample size of 188 children in each treatment group was calculated to provide a probability of 95% or higher of observing at least one severe adverse event in the SMC plus anthelminthic groups, if the true incidence of the severe adverse event was 5% or higher in each treatment group. Haemoglobin (Hb) concentration was also taken into account for calculating the sample size. Given that the mean Hb concentration in the SMC alone group in a previous study [25] was 10.5 *g*/dl, an assumption of a mean Hb concentration of 11.0 *g*/dl in the SMC plus anthelmintic groups, the standard deviation (SD) of the Hb concentration of 1.5 *g*/dl and the mean difference between the treatment groups would be 0.5 *g*/dl, was made. To achieve a power of 90% at 95% statistical significance, a minimum of 189 children per treatment arm was needed. Allowing 10% loss to follow up, approximately 200 children were enrolled into each treatment arm.

### Data management

Field data were collected using a purpose-designed electronic questionnaire by trained research assistants, and these were uploaded on a daily basis on REDCap^®^ (https://www.project-redcap.org/). Laboratory data were managed using Laboratory Information Management System (LIMS) [26]. The field and laboratory data were merged, harmonized, cleaned, and analyzed with STATA^®^ version 18.1 SE (Stata Corp., College Station, TX, USA).

### Statistical analysis

Descriptive statistics such as frequencies and proportions were used to summarize the baseline parasitic parameters and socio-demographical characteristics of the study participants by trial arm. Data were analyzed using an intention-to-treat (ITT) approach. Comparisons were made on the proportions of adverse events (AEs) and the incidence (average number per participant) of reported AEs between the trial arms using Fisher’s exact test and Wald test following Poisson regressions with robust standard errors, respectively. Logistic regressions with robust standard errors were also used to estimate odds ratios. Wald tests were used to assess the evidence of difference in post-randomization odds between trial arms, after controlling for pre-randomization occurrence and baseline characteristics (sex, age group, weight, and malaria and helminth infections’ occurrence). Effect modification between intervention period and trial arm was also investigated using the Wald test. The geometric means of the *P. falciparum* intensities at the pre- and post-intervention periods were estimated and plotted. A univariable Poisson regression with robust standard error and the Wald test were used to compare the pre-intervention parasites intensities across groups, whereas a multivariable Poisson regression including the pre-intervention parasites intensities as a covariate and the Wald test were used to compare the post-intervention parasites intensities.

### Ethical considerations

Ethical approvals for the trial were obtained from the Research Ethics Committees of the London School of Hygiene & Tropical Medicine and the Comité National d’Ethique pour la Recherche en Santé (CNERS) in Senegal. The Participant Information Sheet (PIS) was translated into French and trained field assistants who were native speakers interpreted the consent information in a Senegalese language preferred by the parents/carer-givers. If the parent/carer-giver was not able to read and write in French, a literate adult witness was present throughout the whole consent process and signed and dated the consent form. For children aged ≥ 12 years, positive assent was obtained in addition to the parental consent. All study children were assigned a unique number, which was used as an identifier throughout the study. Participants’ data were securely stored in the dedicated study’s computer devices which were encrypted and password protected. All laboratory results and adverse event data were encoded in an electronic database and stored securely. Only authorized study personnel had access to the study data.

## Results

**Fig. 2:**
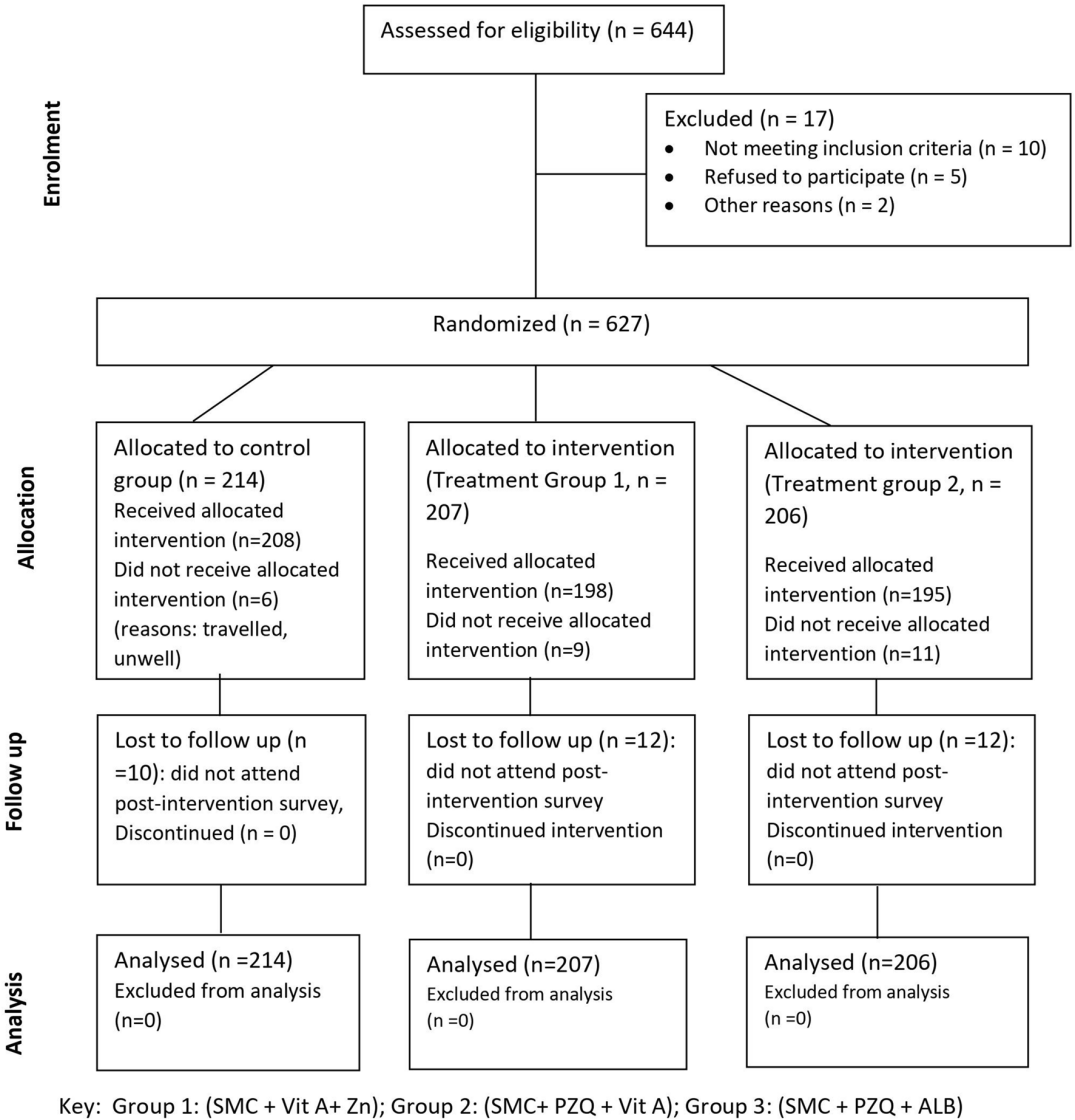
CONSORT diagram showing the flow of the study participants, Saraya, 2022

From 9 to 22 June 2022, 644 children aged 1–14 years living in villages in Saraya district were recruited, and from 18 to 25 November 2022, a post-intervention survey on the enrolled children was conducted. A total of 627 children were randomized into the three groups as follows: control group: SMC + Vitamin A + Zinc = 214; treatment group 1: SMC + PZQ + Vitamin A = 207; treatment group 2: SMC + PZQ + ALB = 206. Approximately two-third of children in each treatment group were aged 5–14 years. Half of the children in each treatment group were female. Figure 2 illustrates the CONSORT flow chart of the recruitment, enrolment, randomization, and follow-up of the study children.

### Pre-intervention findings

Findings at the time of recruitment are shown in Table 2. Wasting was observed in 14% of children (11/214) in the control group, in 10% in the treatment group 1 (7/207) and in 9% in the treatment group 2 (8/206). Stunting was found in one third of children randomized to the three treatment arms: (control group = 33%, treatment group 1 = 33.3%, treatment group 2 = 31.8%). *Plasmodium falciparum* was detected in 8.6% (18/208) of children in the control group, in 7.9% in treatment group 1 (16/203) and in 6.9% in treatment group 2 (14/204). Intestinal protozoans were detected in 62.6% (117/187) of children in the control group, in 56.1% (97/173) in treatment group 1 and in 58.8% (104/177) in treatment group 2. Co-infection with *P. falciparum* and intestinal protozoans were detected in 4.8% (9/187) of children in the control group, in 5.2% (9/173) in treatment group 1 and in 4% (7/177) in treatment group 2. Only one *S. mansoni* infection was detected among control group 0.5% (1/188); two in treatment groups 1 and 2 children: 2% (2/173) and 1.1% (2/178), respectively. Similarly, *A. lumbricoides* and *T. trichiura* were detected in one child in treatment group 2: 0.6% (1/178). No other STH and no *S. haematobium* were detected in children randomized to other treatment groups. Prior to intervention, co-infection with *Plasmodium* spp and intestinal protozoans was 4.8% of the children in the control group (95% CI 2.5–9.0); in 5.2% of children in treatment group 1 (95% CI 2.7–9.7) and in 4% of children in treatment group 2 (95%CI 1.9–8.1).

**Table 2:** Baseline comparison of parasite distribution and socio-demographical characteristics of the study participants, Saraya, 2022

	Control group: (SMC + Vit A + Zn) n = 214 (%)	Treatment group 1: (SMC + PZQ + Vit A) (n = 207 (%)	Treatment group 2: (SMC + PZQ + ALB) n = 206 (%)
Age group			
1–4 years	72 (33.6)	69 (33.3)	65 (31.5)
5–14 years	142 (66.4)	138 (66.7)	141 (68.5)
Gender			
Male	106 (49.5)	101 (48.8)	103 (50.0)
Female	108 (50.5)	106 (51.2)	103 (50.0)
Nutritional status			
WFA			
< -2.5 SD	11 (14.3)		8 (9.3)
> -2.5 SD	66 (85.7)	7 (9.5)	78 (90.7)
HFA		67 (90.5)	
< -2.5 SD	39 (33.0)		40 (31.8)
> -2.5 SD	79 (67.0)	41 (33.3)	86 (68.2)
Hb concentration	199 (100)	191 (100)	192 (100)
< 7 g/dl (severe anaemia)	0 (0.0)	0 (0.0)	0 (0.0)
7–9.9 g/dl (moderate anaemia)	23 (11.6)	21 (10.9)	15 (7.8)
10–10.9 g/dl (mild anaemia)	32 (16.1)	33 (17.1)	29 (15.1)
≥ 11 g/dl (Normal)	144 (72.4)	139 (72.0)	148 (77.1)
*Plasmodium* spp			
Number examined	208	203	204
Infected n (%)	18 (8.6)	16 (7.9)	14 (6.9)
*S. haematobium*			
Number examined	189	173	178
Infected n (%)	0 (0.0)	0 (0.0)	0 (0.0)
*S. mansoni*			
Number examined	188	173	178
Infected n (%)	1 (0.5)	2 (1.2)	2 (1.1)
*Hookworm*			
Number examined	188	173	178
Infected n (%)	0 (0.0)	0 (0.0)	0 (0.0)
*A. lumbricoides*			
Number examined	188	173	178
Infected n (%)	0 (0.0)	0 (0.0)	1 (0.6)
*T. trichiura*			
Number examined	188	173	178
Infected n (%)	0 (0.0)	0 (0.0)	1 (0.6)
Intestinal protozoans*			
Number examined	187	173	177
Infected n (%)	117 (62.6)	97 (56.1)	104 (58.8)
*Plasmodium* + STH + Schisto			
Number examined	188	173	178
Infected n (%)	0 (0.0)	0 (0.0)	0 (0.0)
*Plasmodium* + *Intestinal protozoans*			
Number examined	187	173	177
Infected n (%)	9 (4.8)	9 (5.2)	7 (4.0)

Key: *WFA* Weight for age, *WFH* weight for height, *HFA* height for age, *STH* Soil transmitted helminths = Hookworm + *A.lumbricoides* + *T.trichiura*, Schisto** = ***S.haematobium* + *S.mansoni* *Intestinal protozoans are a group of diseases caused by one or more species of protozoa and helminths e.g.: *Entamoeba coli, Giardia intestinalis, Blastocystis hominis, Endolimax nana*

### Safety results

**Fig. 3:**
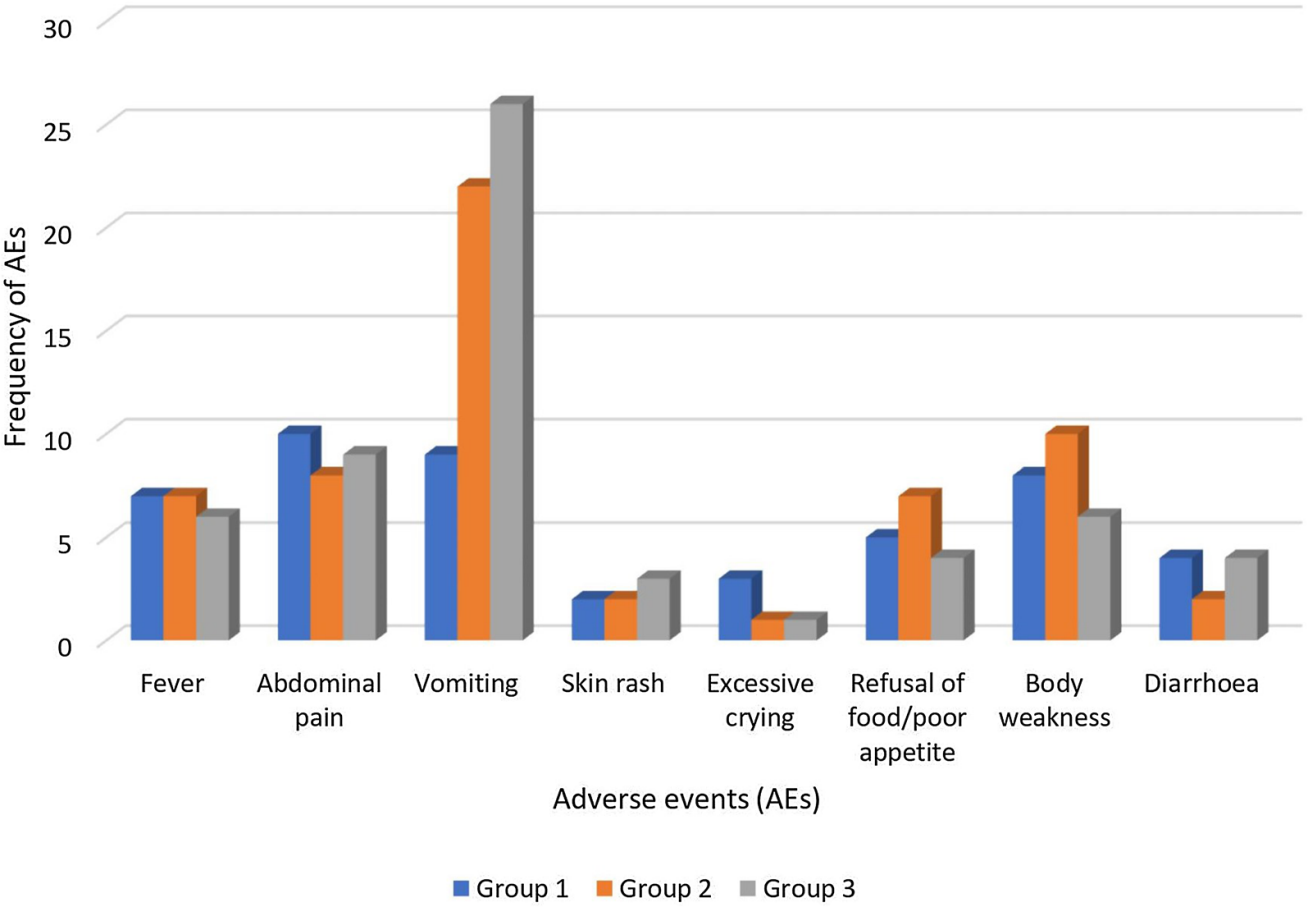
Distribution of adverse events across the treatment arms in the first 6 days post-administration of SMC and anthelminthic drugs. *Key: Group 1* = *control group, Group 2* = *Treatment group 1, Group 3* = *Treatment group 2*

Figure 3 showed the distribution of adverse events over the first 6 days post-administration of the study drugs. Vomiting was observed in 12.6% (26/206) of children randomized to treatment group 2, in 10.6% (22/207) of those in treatment group 1 and in 4.2% (9/214) of children in the control group (p = 0.005). The episodes of vomiting across the three treatment groups were transient and of mild intensity. Fever was reported in 3.3% (7/214) of the children in the control group, in 3.4% (7/207) of those in treatment group 1 and in 2.9% (6/206) in treatment group 2. Abdominal pain, skin rash, and refusal of food/poor appetite were observed across the control group, treatment groups 1 and 2, 4.7% vs 3.9% vs 4.4%; 0.9% vs 1.0% vs 1.5%; and 2.3% vs 2.4% vs 1.9%, respectively. Similar patterns were observed when the incidence i.e. number of reported AEs per 100 participants (95% CI) was calculated, with vomiting observed in 4% (95% CI 1.9–8.0) of children in the control group, 11% (95% CI 7–16) in the treatment group 1; and in 13% (95% CI 8–18) in treatment group 2 (p = 0.01). Further information on safety events is provided in Table 3.

**Table 3:** Summary of the frequency and grade of solicited and unsolicited adverse events

	Control group	Treatment group 1	Treatment group 2	P-value***
Number of participants, n (%)	214 (100)	207 (100)	206 (100)	–
Proportion of participants who experienced at least one AEs, n (%) [1]				
Overall	19 (8.9)	30 (14.5)	36 (17.5)	0.03
Fever	7 (3.3)	7(3.4)	6 (2.9)	0.99
Abdominal pain	10 (4.7)	8 (3.9)	9 (4.4)	0.94
Vomiting	9 (4.2)	22 (10.6)	26 (12.6)	0.005
Skin rash	2 (0.9)	2 (1.0)	3 (1.5)	0.81
Excessive crying	3 (1.4)	1 (0.5)	1 (0.5)	0.63
Refusal of food/poor appetite	5 (2.3)	7 (3.4)	4 (1.9)	0.67
Body weakness	8 (3.7)	10 (4.8)	6 (2.9)	0.59
Diarrhoea	4 (1.9)	2 (1.0)	4 (1.9)	0.73
Total number of reported AEs ^a^	53	63	63	–
Incidence *i.e.* number of reported AEs per 100 participants (95% CI)^a^				
Overall	25 (19–32)	30 (23–39)	31 (24–39)	0.81^#^
Fever	3.3 (1.3–6.7)	3.4 (1.4–7.0)	3.9 (1.7–7.7)	0.96^#^
Abdominal pain	5.1 (2.6–9.2)	4.8 (2.3–8.9)	4.4 (2.0–8.3)	0.94^#^
Vomiting	4.2 (1.9–8.0)	11 (7–16)	13 (8–18)	0.01^#^
Skin rash	1.4 (0.3–4.1)	1.0 (0.1–3.5)	1.9 (0.5–5.0)	0.75^#^
Excessive crying	1.4 (0.3–4.1)	0.5 (0.01–2.7)	0.5 (0.01–2.7)	0.51^#^
Refusal of food/poor appetite	3.3 (1.3–6.7)	3.9 (1.7–7.6)	1.9 (0.5–5.0)	0.54^#^
Body weakness	4.2 (1.9–8.0)	5.3 (2.7–9.5)	2.9 (1.1–6.3)	0.51^#^
Diarrhoea	1.9 (0.5–4.8)	1.0 (0.1–3.5)	2.4 (0.8–5.7)	0.57^#^
Number of SAEs reported	0	0	0	–
Number of participants with SAEs, n (%) [2]	0 (0.0)	0 (0.0)	0 (0.0)	–
Severity of all AEs**				
Grade 1 = mild	18 (94.7)	28 (93.3)	35 (97.2)	0.38
Grade 2 = moderate	0 (0.0)	2 (6.7)	1 (2.8)	
Grade 3 = severe	1 (5.3)	0 (0.0)	0 (0.0)	
Grade 4 = life threatening	0 (0.0)	0 (0.0)	0 (0.0)	

^[1]^ Participants who experienced one or more AEs or SAEs are counted only once

^[2]^ Participants are counted only once within a particular severity grade or relatedness category

^a^ Accounts for multiple episodes

^*^ Percentages based on number of AEs reported for each treatment group

^**^ Percentages based on N for each treatment group

^***^ Fisher’s exact test. ^#^Wald test based on Poisson’s robust standard errors

### Post-intervention findings

**Fig. 4:**
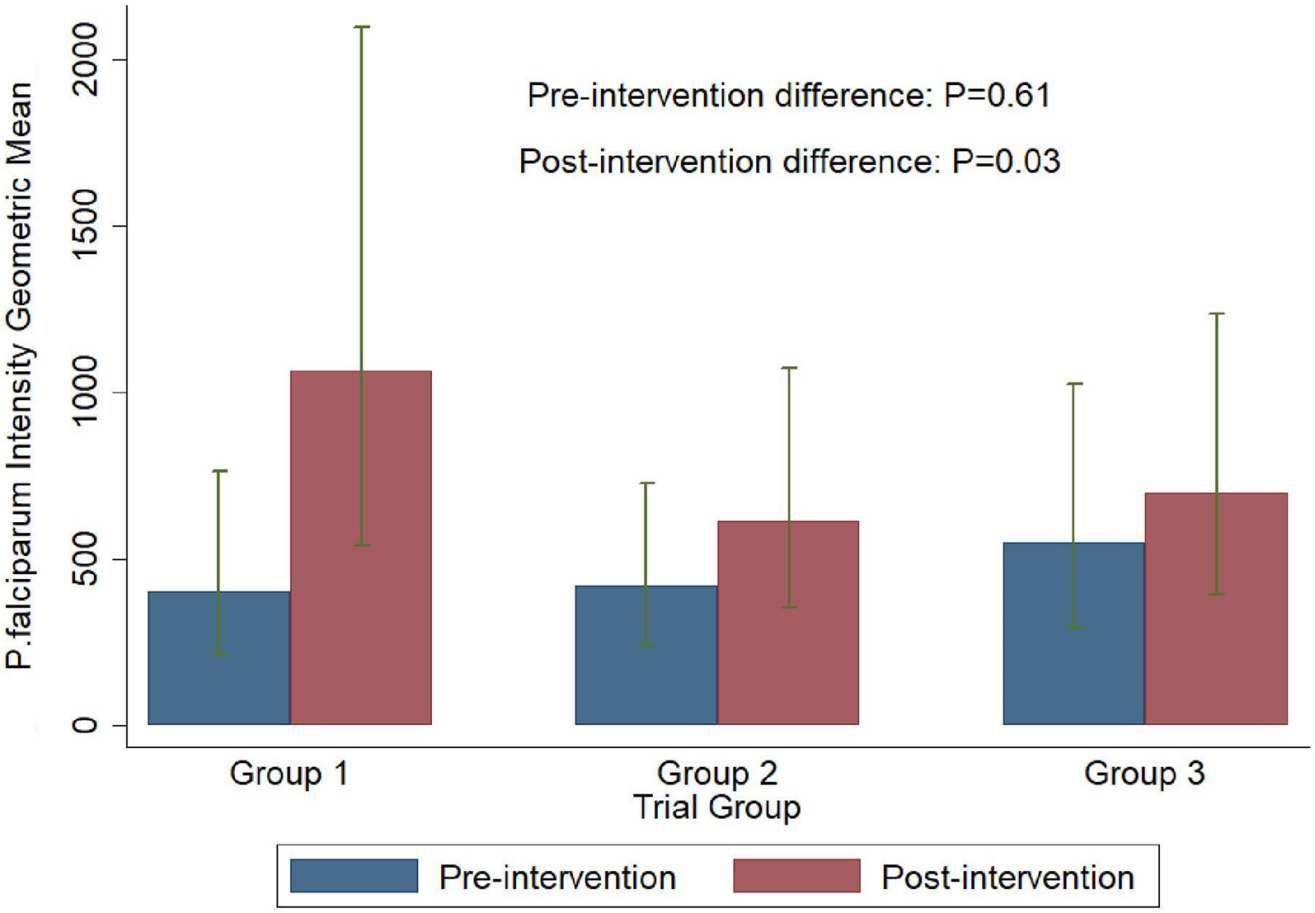
Pre-and post-intervention *P. falciparum* Intensity Geometric Mean and 95% confidence intervals across the three study groups. *Key: Group 1* = *control group, Group 2* = *Treatment group 1, Group 3* = *Treatment group 2*

Five months post-intervention, the prevalence of *Plasmodium* spp was 8.9% (95% CI 5.7–13.5) among control group children, 12.1% (95% CI 8.3–17.3) in treatment group 1 children and 11.2% (95% CI 7.5–16.3) in treatment group 2 children. The odds ratio for malaria infection of children in the treatment group 1 compared with those in the control group (OR: 1.45; 95% CI 0.71–2.96), and the odds ratio of children in treatment group 2 compared with those in the control group 1 (OR: 1.21; 95% CI 0.59–2.57) did not reach statistical significance. Post-intervention, the intensity of *P. falciparum* parasitaemia was highest among children in the control group, and differed significantly between groups (p = 0.03) (Fig. 4).

Post-intervention, the prevalence of *S. mansoni* remained very low in all three groups. Also, the prevalence of intestinal protozoans increased slightly to 60% (95% CI 52.6–66.9) in the control group; to 65.9% (58.4–72.6) in treatment group 1 and to 66.4% (59.2–73.0) in treatment group 2. Higher prevalence rates were observed post-intervention, with the co-infection rate of *P. falciparum* spp and intestinal protozoans at 5.6% (95% CI 3.0–10.0) in the control group; 10% (95% CI 6.3–15.5) in treatment group 1 and 10.6% (95% CI 6.9–16.1) in treatment group 2. However, the difference in the prevalence between the groups did not reach statistical significance (p = 0.40). The risk of having *Plasmodium*-intestinal protozoan co-infection did not differ significantly between children in treatment group 1 and control group (OR = 1.96, 95% CI  0.79–4.82) and between treatment group 2 and control group (OR = 1.95, 95% CI 0.79–4.78) (Table 4). The risk of developing severe anaemia between treatment group 1 and the control group was 0.81 (95% CI 013–5.00) and was 1.78 (95% CI 0.38–8.27 (p = 0.63) between treatment group 2 and the control group (Table 4). Prior to intervention, the geometric mean value of *P. falciparum* parasite density was highest among children in treatment group 2, but this did not differ significantly between the groups (p = 0.63). Post-intervention, the geometric mean value was highest among children in the control group, with a significant difference across the arms (p = 0.03) (Fig. 4).

**Table 4:** Effect of interventions on prevalence (%) of malaria-helminth co-infection among study participants, Saraya, 2022

	Pre-intervention Prevalence 95% CI			Post-intervention Prevalence 95% CI			Odds ratio OR 95% CI	Odds ratio OR 95% CI	P value*	P value**
	Control group	Treatment Group 1	Treatment Group 2	Control Group	Treatment Group 1	Treatment Group 2	Treatment Group 1 vs control group, Post-intervention	Treatment Group 2 vs control group, Post-intervention		
*Plasmodium* spp										
* P. falciparum*	8.4 (5.4–13.0)	7.7 (4.8–12.3)	6.8 (4.1–11.2)	8.9 (5.7–13.5)	12.1 (8.3–17.3)	11.2 (7.5–16.3)	1.45 (0.72–2.91)	1.24 (0.60–2.54)	0.58	0.33
***P. malariae	8.4 (5.4–13.0)	6.8 (4.0–11.1)	6.3 (3.7–10.6)	8.4 (5.4–13.0)	11.6 (7.9–16.7)	10.7 (7.1–15.7)	1.45 (0.71–2.96)	1.24 (0.59–2.57)	0.59	0.22
*Schistosomiasis*										
* S. haematobium*	0.5 (0.07–3.7)	1.1 (0.3–4.5)	(0.3–4.4)	0.5 (0.08–3.8)	1.2 (0.3–4.6)	1.1 (0.3–4.3)	–	–		–
***S. mansoni	0.5 (0.07–3.7)	1.1 (0.3–4.5)	1.1 (0.3–4.5)	0.5 (0.08–3.8)	1.2 (0.3–4.6)	1.1 (0.3–4.3)				
STH										
***Hookworm	0	0	1.1 (0.3–4.4)	0.6 (0.08–3.8)	0.6 (0.08–4.1)	0	–	–		–
***A. lumbricoides										
***T. trichiura										
Intestinal protozoans	62.6 (55.4–69.2)	56.1 (48.6–63.3)	58.9 (51.3–65.8)	60.0 (52.6–66.9)	65.9 (58.4–72.6)	66.4 (59.2–73.0)	1.25 (0.78–2.01)	1.37 (0.86–2.19)	0.40	0.30
Co-infection										
* Plasmodium* + intestinal protozoans	4.8 (2.5–9.0)	5.2 (2.7–9.7)	4.0 (1.9–8.1)	5.6 (3.0–10.0)	10.0 (6.3–15.5)	10.6 (6.9–16.1)	1.96 (0.79–4.82)	1.95 (0.79–4.78)	0.26	–
Hb (g/dl)										
(severe anaemia vs moderate/mild/ normal)	0	0	0	1.5 (0.5–4.7)	1.1 (0.3–4.3)	2.1 (0.8–5.5)	0.81 (0.13–5.00)	1.78 (0.38–8.27)	0.63	0.83

^*^Wald test assessing evidence of difference in post-randomization prevalence between trial arms, after controlling for pre-randomization occurrence of outcome and baseline characteristics (sex, age group, weight, and other infections’ occurrence). ** Effect modification test between intervention period and trial arm. ***Number very low to enable statistical comparisons

## Discussion

This randomized controlled trial demonstrated that the integration of MDA for schistosomiasis and STH with SMC drugs is feasible logistically, with a high retention of study participants 5 months after the administration of the drugs to study children. This is the first study proving the feasibility of delivering MDA for schistosomiasis and STH through an SMC platform. This finding has provided evidence to boost the public health recommendations for combined malaria and helminth control and reinforces the empirical evidence that the future direction of health care systems in developing countries should be comprehensive health management rather than vertical management of a single disease. The delivery method adopted for the integrated strategy, involving house-to-house visits, contributed to the logistical feasibility and high retention. Similarly, using local health workers who understood the study communities and their cultures made the combined approach to gain the trust and acceptance of the parents of the study children. More importantly, the collaborative support provided by the Senegal national malaria and NTD control programmes made the implementation of the integrated strategy logistically feasible. These factors have been documented to be effective strategies in the vertical implementation of MDA and SMC in most African settings [25, 27, 28]. Therefore, replicating the strategies in implementing an integrated approach in malaria-helminth co-endemic countries will optimize SMC and NTD control programmes.

The administration of the anthelminthic drugs (PZQ and ALB) and SMC drugs (SPAQ) on separate days (24 h apart) provided an opportunity to objectively assess the tolerability of the different drug regimen. Vomiting was the most common adverse events reported among children in the treatment groups 2 and 3 who were randomized to receive praziquantel with SMC drugs. This adverse event was transient, mild to moderate in intensity and did not prevent the children from taking SMC drugs in the subsequent days. Nevertheless, the high incidence of vomiting among the children in these treatment groups may pose a major challenge to the uptake and acceptability of the integrated malaria-helminth preventive treatment approach by the children and their parents. This finding resonated with the feedback obtained through qualitative interviews conducted among the parents/caregivers of the children enrolled in this trial, during which they expressed concerns about the bitter taste, smell, and vomiting-induced effect of praziquantel [29]. Previous studies have also documented the increased incidence of these adverse events with praziquantel [28, 30]. Amodiaquine, which is a partner drug in the SMC regimen, has also been reported to cause nausea and vomiting in children [31], but this was not a significant issue in this study. Given that this adverse event is dose-dependent, administration of accurate and age-appropriate dosing of the drug to each child was ensured. In addition, to mask the bitter taste of the amodiaquine and praziquantel and to improve their palatability, the drugs were re-purposed by the study pharmacists by adding a measured amount of granulated sugar. This strategy is consistent with good pharmaceutical practice of adding sweetening agents to mask the bitter taste and make partially dissolved drugs more palatable [32]. Nevertheless, the introduction of a child-friendly, flavoured formulations of praziquantel in the coming year will significantly address the side effects of the bitter taste, nausea and vomiting associated with praziquantel [33, 34].

Other adverse events such as fever, abdominal pain, skin rash, refusal of food/poor appetite and diarrhoea were similar in frequency across the three groups and were generally mild and transient. Although the prevalence of *P. falciparum* in the intervention groups was higher than in the SMC alone group in the post-intervention assessment, malaria parasite density was much higher in the control group than the intervention arms. Children who received praziquantel and SMC drugs had a lower risk of developing severe anaemia than their counterparts who received SMC drugs alone. This is contrary to the observations among the study children who received albendazole, praziquantel and SMC drugs. This could be explained by the extremely low prevalence of schistosomiasis and STH which made an impact assessment difficult.

This study had some limitations. Given that SMC is a standard preventive treatment for malaria in the study area, a control group for SMC could not be included. However, historical data showed that a much higher prevalence of malaria and lower Hb concentration would have been reported at the end of the transmission season without SMC in the study area [25, 35]. Therefore, the study findings support the evidence that SMC worked well in ensuring that the prevalence of malaria did not rise significantly, post intervention. As highlighted above, it was not possible to assess the impact of the intervention on schistosomiasis and STH because their prevalence was extremely low in the study area. To ensure that the prevalence of the malaria-helminth co-infection was robust enough to support the implementation of this study, baseline surveys in two epidemiologically distinct communities in Senegal were conducted in the preceding year, the findings of which shaped the selection of the site for the current trial. Although a low prevalence of malaria, STH and schistosomiasis was found in many villages during the surveys [7], villages with a high prevalence of malaria, schistosomiasis and STH were carefully selected for this trial. This study was also implemented in partnership with the Senegal malaria and NTD control programmes, to ensure that the timing of NTD and SMC campaigns aligned with the study implementation timelines. It is, therefore, difficult to explain the reasons for the extremely low prevalence of schistosomiasis and STH, contrary to the findings in the villages selected for this trial about 10 months earlier [7]. There was also no intervention or rounds of MDA implemented during the gap period between initial survey and the trial. Nevertheless, this study established that MDA for schistosomiasis and STH may be unnecessary in this community at the current time. Periodic impact assessments by national or regional control programmes could help in deciding when MDA programmes are no longer needed in the study area, having attained the goal of eliminating morbidity associated with STH (i.e., elimination as a public health problem, defined as a moderate-to-heavy intensity infection prevalence of < 2%) [36].

Consistent with the findings of the preceding surveys [7], a very high prevalence of intestinal protozoans across the three treatment groups was observed and this is reflected in the co-infections with *P. falciparum*, with no observable impact of the interventions. The incidental findings of a high prevalence of intestinal protozoans in a community where STH and schistosomiasis have been nearly eliminated calls for a strong consideration of an addition of appropriate medications to address the silent burden of intestinal protozoans in the study population. A similar effort is currently underway to develop and evaluate a combination of effective drug treatments against all five species of STH [37].

## Conclusion

This study has demonstrated that integration of MDA for helminths with SMC was feasible and safe among Senegalese children living in a low transmission setting of malaria and helminth co-endemicity. These findings underscore the important role of context, programme partnerships and community involvement in overcoming the logistical challenges of integrating two vertical control programmes involving delivery of MDA for helminths through SMC platform. The findings on feasibility and safety of the combined approach support further evaluation in co-endemic settings with a high prevalence of all pathogens of interest. This would address the gaps experienced in this study and could pave the way to achieving equitable progress in the effective control and elimination of malaria and helminths, in line with the 2030 WHO targets [36, 38].

## Data Availability

All data generated or analysed during this study are included in this published article.

## Abbreviations

AE: Adverse events
ALB: Albendazole
CI: Confidence interval
MDA: Mass drug administration
NMCP: National Malaria Control Programme
NTD: Neglected tropical diseases
PCR: Polymerase Chain Reaction
PZQ: Praziquantel
SAE: Serious adverse events
SMC: Seasonal malaria chemoprevention
SPAQ: Sulphadoxine pyrimethamine and Amodiaquine
STH: Soil transmitted helminths
